# Circulating microRNAs Reveal Time Course of Organ Injury in a Porcine Model of Acetaminophen-Induced Acute Liver Failure

**DOI:** 10.1371/journal.pone.0128076

**Published:** 2015-05-27

**Authors:** Luisa A. Baker, Karla C. L. Lee, Carolina Palacios Jimenez, Hatim Alibhai, Yu-Mei Chang, Pamela J. Leckie, Rajeshwar P. Mookerjee, Nathan A. Davies, Fausto Andreola, Rajiv Jalan

**Affiliations:** 1 Department of Clinical Science and Services, Royal Veterinary College, Hatfield, Hertfordshire, United Kingdom; 2 Liver Failure Group, University College London Institute for Liver and Digestive Health, University College London Medical School, Royal Free Hospital, London, United Kingdom; University of Medicine, Greifswald, Germany, GERMANY

## Abstract

Acute liver failure is a rare but catastrophic condition which can progress rapidly to multi-organ failure. Studies investigating the onset of individual organ injury such as the liver, kidneys and brain during the evolution of acute liver failure, are lacking. MicroRNAs are short, non-coding strands of RNA that are released into the circulation following tissue injury. In this study, we have characterised the release of both global microRNA and specific microRNA species into the plasma using a porcine model of acetaminophen-induced acute liver failure. Pigs were induced to acute liver failure with oral acetaminophen over 19h±2h and death occurred 13h±3h thereafter. Global microRNA concentrations increased 4h prior to acute liver failure in plasma (*P*<0.0001) but not in isolated exosomes, and were associated with increasing plasma levels of the damage-associated molecular pattern molecule, genomic DNA (*P*<0.0001). MiR122 increased around the time of onset of acute liver failure (*P*<0.0001) and was associated with increasing international normalised ratio (*P*<0.0001). MiR192 increased 8h after acute liver failure (*P*<0.0001) and was associated with increasing creatinine (*P*<0.0001). The increase in miR124-1 occurred concurrent with the pre-terminal increase in intracranial pressure (*P*<0.0001) and was associated with decreasing cerebral perfusion pressure (*P*<0.002).

**Conclusions:**

MicroRNAs were released passively into the circulation in response to acetaminophen-induced cellular damage. A significant increase in global microRNA was detectable prior to significant increases in miR122, miR192 and miR124-1, which were associated with clinical evidence of liver, kidney and brain injury respectively.

## Introduction

Drug-induced liver injury accounts for more than 50% of acute liver failure (ALF) cases with the largest number of cases being attributable to acetaminophen (APAP) [1]. ALF is a rare but catastrophic condition that is caused by massive hepatocyte necrosis and characterised by hepatic and extrahepatic organ failure, notably of the kidney and brain [2]. Acute kidney injury is reported to occur in 80% of patients with APAP-induced ALF and is associated with worse outcomes [3,4], whilst intracranial hypertension, caused by brain oedema, accounts for 20–25% of deaths due to ALF [5]. As a result the different predictive scoring systems used to determine the requirement for liver transplantation, including King's College Criteria, APACHE II, SOFA score, MELD and Clichy-Villejuif criteria, all utilise a marker of liver function or coagulopathy, and at least one of creatinine or encephalopathy grade [6,7]. The prognosis and requirement for liver transplantation remain difficult to predict [8] and partly as a consequence of this, the death rate of patients with APAP-induced ALF is approaching 30% [1]. Gaining a more detailed understanding of the time course of organ injury in ALF may hold the key to better prediction of outcomes, identification of therapeutic windows, earlier targeted organ-specific interventions and therefore improved patient survival in ALF.

Both damage-associated molecular pattern molecules (DAMPs) and microRNA (miRNA) are released into the circulation following tissue injury [9,10] and have been shown to increase in plasma samples during ALF [11,12]. Genomic DNA is an established DAMP, which is released following tissue injury in ALF, and is involved in the activation of the immune response via toll-like receptor-9 [13]. MiRNA are short (ca. 22 nucleotides), non-coding strands of RNA that function as post-transcriptional regulators and are highly conserved between species [14]. RNA strands may also activate the immune response via toll-like receptor-7 [9]. In addition, miRNA have been shown to be stable in blood samples under extreme conditions, making them good candidates for biomarkers [15–17].

Previous studies have considered the potential use of miRNA species as biomarkers in ALF. In a mouse model of APAP-induced acute liver injury (ALI), plasma miR122 and miR192 increased in a time- and dose-dependent manner in parallel with increasing alanine transaminase (ALT) [12]. These findings were replicated in a study of human patients with ALI, with both circulating miR122 and miR192 being increased in patients with APAP-induced ALI but only miR122 correlating with peak ALT concentrations [18]. Further work in APAP overdose patients demonstrated that plasma miR122 levels at presentation to hospital correlated with peak ALT and peak international normalised ratio (INR) during the period of hospitalisation and that in patients with normal ALT at presentation, miR122 levels were predictive of development of ALI and more sensitive than ALT for identifying ALI in patients presenting within eight hours of APAP overdose [19]. John *et al* [20] extended these findings by demonstrating that serum and liver tissue levels of miR122 were higher in ALF patients that spontaneously recovered compared to those that required liver transplantation, suggesting that miR122 levels may be predictive of recovery from ALF.

Whilst circulating miR192 has been shown to increase following APAP-induced ALI in both mice and humans [12,18], the lack of correlation with ALT makes its organ of origin uncertain. MiR192 is considered kidney-specific [21] and is highly expressed in the proximal convoluted tubule [22], which has been shown to be the site of injury associated with acute kidney injury in APAP-induced ALF [4]. MiR124-1 has been shown to be enriched in the brain [23,24], released into the plasma following brain injury in a rat model of stroke [25], and a predictor of neurological outcome in humans following cardiac arrest [26]. Changes in circulating levels of miR124-1 and its relationship with brain injury during the progression of ALF have yet to be investigated.

In view of the potential dual role of elevated circulating miR122 both as an early predictor of onset of ALI in APAP toxicity [19], as well as a predictor of survival in ALF [20], characterisation of the temporal changes in plasma miR122 levels with disease progression may aid understanding of its role in ALF. Furthermore, as ALF is a dynamic disease, knowledge of the temporal changes in plasma levels of a potential biomarker is useful when considering results from a single time point. In addition, since tissue-specific miRNAs have been identified for the major organs affected in ALF [21,23], miRNAs offer the potential to investigate not just the liver injury occurring during ALF, but also the significant co-morbidities of kidney and brain injury.

Thus, the present study investigated the time course of changes in plasma levels of global miRNA and tissue-specific miRNA using samples from a reproducible and clinically relevant porcine model of APAP-induced ALF that allows monitoring of clinical and analytical parameters during the evolution of ALF, from the point of APAP overdose through ALI to death [27].

## Materials and Methods

### Porcine model of APAP-induced ALF

Banked samples from our previously published study of a porcine model of ALF induced with oroduodenal APAP dosing, supported as in a human intensive care setting [27] were used. Briefly, six APAP and three control pigs (30-40kg) were maintained under total intravenous anaesthesia with ketamine, midazolam and fentanyl. Clinical onset of ALF was documented by monitoring INR, which increased to greater than 3, 19h ± 2h after the onset of APAP dosing following a total APAP dose of 59.6g ± 10.5g. The time at which INR exceeded 3 will be referred to hereafter as the point of ‘ALF’ for ease of description. At ALF, two units of porcine fresh frozen plasma were given and continuous renal replacement therapy (PRISMA HF1000, Gambro Dialysatorium GmbH, Rostock, Germany) was initiated. Thereafter, all APAP animals developed multi-organ failure, characterised by intracranial hypertension, hyperammonaemia, cardiovascular collapse, elevation in creatinine and metabolic acidosis, and died from non-recoverable cardiorespiratory arrest after a further 13h ± 3h. The biochemical and clinical progression of ALF in these pigs has been previously described. Control animals underwent sham induction to ALF over 20h with water instead of APAP and were maintained for a further 20h. Control animals were managed using the same protocols as the APAP group, including initiation of continuous renal replacement therapy (PRISMA HF1000) at ALF. All animal procedures were conducted under a project license approved by the UK Home Office and in strict accordance with the Animals (Scientific Procedures) Act 1986.

Plasma samples had been obtained every 4h from the onset of APAP dosing, at the point of ALF and every 4h until death. Dialysate samples had been obtained every 4h after the initiation of continuous renal replacement therapy at ALF. Tissue biopsies had been collected at death. All samples were stored at -80^°^C.

### miRNA

#### Isolation and quantification

RNA was isolated from aliquots of plasma and dialysate, which had not been previously thawed, exosomes and tissue samples and the small RNA-enriched fraction (including miRNA) was separated from larger RNAs using the miRNeasy Mini kit (Qiagen, Hilden, Germany) according to the manufacturer’s protocol. The quantity and quality of the isolated small RNA and miRNA was assessed using Small RNA chips on the Agilent 2100 Bioanalyzer (Agilent Technologies, Santa Clara, CA).

#### Establishing a porcine endogenous control

Following a literature search, three potential endogenous controls were identified, snRNA:U6, miR26a and miR191. Their TaqMan MicroRNA Assays (Applied Biosystems, Foster City, CA; S1 Table) were assessed for amplification efficiency and validated for use in porcine samples, and they were tested for stability with progression of ALF [28–32]. Of the three potential endogenous controls, miR26a had threshold cycles (C_t_s) closest to the target miRNA species and was the most stable throughout ALF (Table 1 and S1 Fig). As a result miR26a was used as the endogenous control for the specific miRNA assays.

**Table 1:** Assessment of potential endogenous controls, snRNA:U6, miR26a and miR191, for use in porcine studies of ALF, using miR122 as the target miRNA.

Gene	Raw Ct (Mean ± SD)	Normfinder (Stability value)	BestKeeper (SD of CPs)	geNorm (M-value ± CV)	Delta Ct (Mean SD)	Composite Rank
**miR26a**	29.52 ± 0.19	0.019	0.18	1.319 ± 0.163	1.825	**1**
**miR191**	24.48 ± 1.42	0.069	1.19	1.816 ± 0.588	2.517	**2.25**
**snRNA:U6**	34.10 ± 1.89	0.051	1.59	2.115 ± 1.078	2.922	**2.75**

Potential endogenous controls were quantified by qRT-PCR in plasma samples from acetaminophen-treated pigs (n = 3, raw Ct). The stability of the potential endogenous controls with progression of ALF was assessed using the following software packages: Normfinder [28]; BestKeeper [29]; geNorm [30,31]; and Delta Ct method [32]. The results from these analyses were used to form the composite rank to determine the final choice of endogenous control. For a graphical representation of the stability of the endogenous controls see S1 Fig.

Ct, threshold cycle; SD, standard deviation; CPs, crossing points; M-value, gene stability value; CV, coefficient of variance

#### Quantification of specific miRNA species

Specific miRNA species (miR122, miR192 and miR124-1) and miR26a were quantified by reverse transcription quantitative polymerase chain reaction (qRT-PCR) starting with an input miRNA of 0.25ng for miR122 and miR192, and 0.5ng for miR124-1, and using TaqMan MicroRNA Assays (S1 Table) with TaqMan MicroRNA Reverse Transcription Kit and TaqMan Universal PCR Master Mix, No AmpErase UNG on the 7500 Fast Real-Time PCR system (all Applied Biosystems). All specific miRNA species were quantified in the plasma and dialysate, and the relevant tissue-specific miRNA along with the endogenous control were quantified in each of the liver, kidney and brain tissues. It was not possible to quantify miR124-1 at the +12h time point due to a lack of available plasma.

### Exosomes

#### Precipitation

ExoQuick exosome precipitation solution (EXOQ5A, System Biosciences, Mountain View, CA) was mixed with 100μl of plasma, which had not been previously thawed, and the exosome-rich fraction was precipitated according to the manufacturer’s protocol. (Levels of exosomal and protein-bound miRNA have been shown to be stable during prolonged cold storage (-20^°^C and below) but not with repeated freeze-thaw cycles [33].) The exosome pellets were then lysed to isolate either small RNAs using the miRNeasy Mini kit (see above; Qiagen, Hilden, Germany) or proteins using 1X RIPA buffer (25mM Tris-HCl, pH7.6, 150mM NaCl, 1% NP-40, 1% sodium deoxycholate, 0.1% SDS plus a cocktail of protease inhibitors; Sigma, St. Louis, MO).

#### Protein quantification and Western blotting

After lysis in RIPA buffer, exosome protein quantification was carried out using the MicroBCA method (Pierce Protein Biology Products, Thermo Fisher Scientific, Waltham, MA); 20 μg of sample was then resolved in 4–12% Bis-Tris NuPage gels (Invitrogen, Carlsbad, CA). Proteins were then transferred onto PVDF membranes. Membranes were blocked and incubated with primary antibodies [HSP (heat shock protein) 70 (R&D systems, Minneapolis, MN), CD9A1 (System Biosciences)]. The membranes were washed and incubated again with a horseradish peroxidase-conjugated specific secondary antibody. The bound antibody was detected using an enhanced chemiluminescence reagent.

### Quantification of genomic DNA levels

Plasma genomic DNA levels were measured with the Cell Death Detection ELISA Plus (Roche, Basel, Switzerland) using 20μl plasma and following the manufacturer’s instructions. To ensure consistency between the two plates, three samples were run on both plates, a linear regression was plotted and the two plates were assimilated.

### Data analysis and statistics

#### Clinical data

In this study, recordings of INR, creatinine, cerebral perfusion pressure (CPP), bicarbonate therapy, noradrenaline therapy and albumin were used as markers of progression of liver injury, kidney injury, brain injury, acid-base imbalance, cardiovascular stability and vascular permeability respectively. CPP was calculated as mean arterial pressure minus intracranial pressure (ICP). Collection and description of this data has been reported previously [27].

#### miRNA data

For the qRT-PCR data the threshold line was established from pilot studies and standardised across all experiments. C_t_s from three technical replicates were summarised as mean values for each miRNA in a sample. The raw C_t_ means were placed relative to both the mean C_t_ of the endogenous control in the same sample (ΔC_t_) and the mean ΔC_t_ of the control animals at that time point (ΔΔC_t_). qRT-PCR data were therefore summarised and displayed as relative expression (2^-ΔΔCt^), corrected to μl of plasma where appropriate.

#### Statistics

All data were summarised as mean ± SE. Linear mixed effects models were used for all analyses, and the first degree auto-regressive (co)variance structure was used to account for the correlation between repeated measures. The fixed effects (β) and confidence interval (CI) of each covariate were estimated and represented on their respective log and semi-log graphs. Significance was set at the 5% level. Statistical analyses were performed using SPSS software version 21 for Windows (IBM, Armonk, NY).

## Results

### Plasma global miRNA levels increase with onset of ALF and are associated with disease progression

The temporal changes in the six markers of disease progression used in this study are demonstrated in S2 Fig. Briefly, INR was only monitored from the onset of APAP dosing until ALF and increased from baseline at 16h after the onset of APAP dosing, continuing to rise to ALF (*P* < 0.0001). Creatinine was elevated from 16h after the onset of APAP dosing until death (*P* < 0.0001). CPP began to decrease at ALF and continued to fall until death (*P* < 0.0001). Acid-base imbalance reflected by bicarbonate therapy was different from controls from ALF to 8h thereafter (*P* < 0.0001). Reduced cardiovascular stability was reflected by increased noradrenaline therapy requirements in APAP pigs from ALF until death (*P* < 0.0001). Reduced circulating albumin concentration from 16h after the onset of APAP dosing until death (*P* < 0.0001) reflected increased vascular permeability.

**Fig 1:**
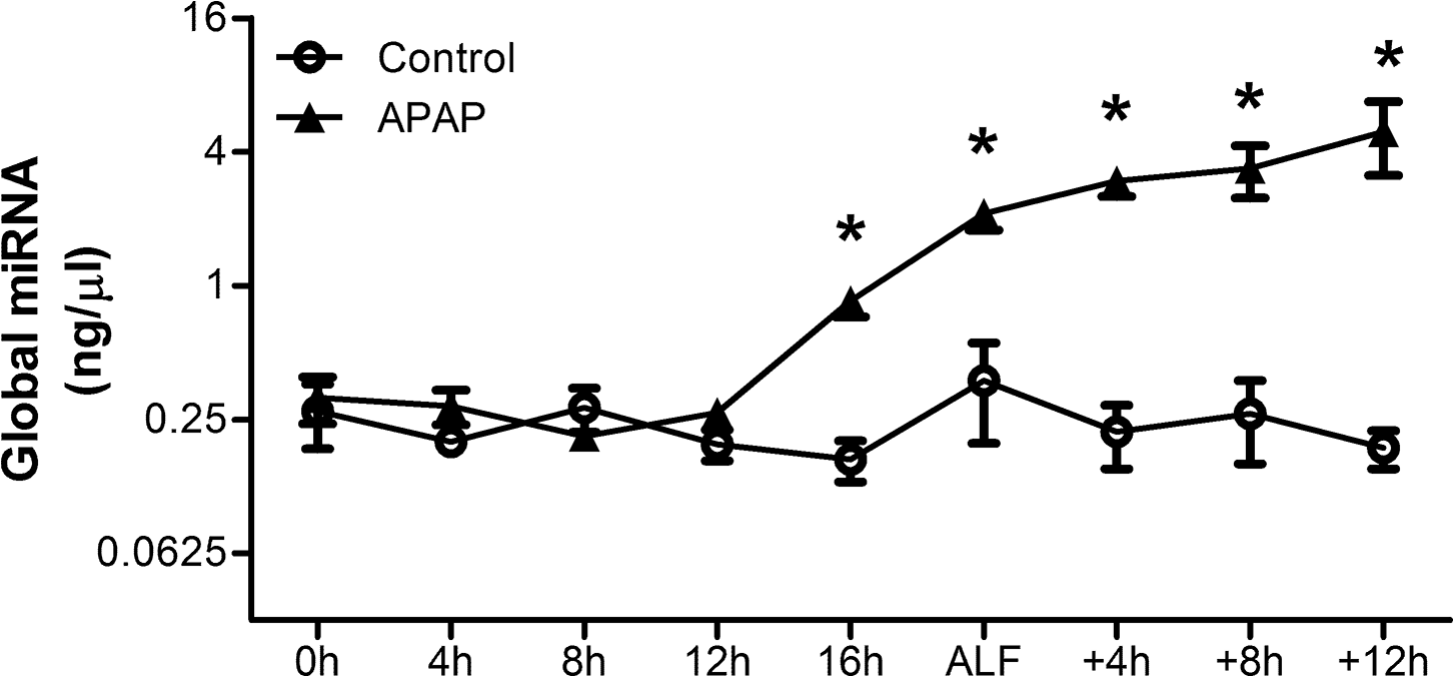
Plasma global miRNA concentrations increase in APAP-treated animals. Plasma global miRNA concentrations were measured every 4h from the onset of dosing until death for APAP-treated pigs (n = 6) and controls (n = 3). Values are means ± SE; * *P* < 0.05 vs. baseline at -20h.

**Fig 2:**
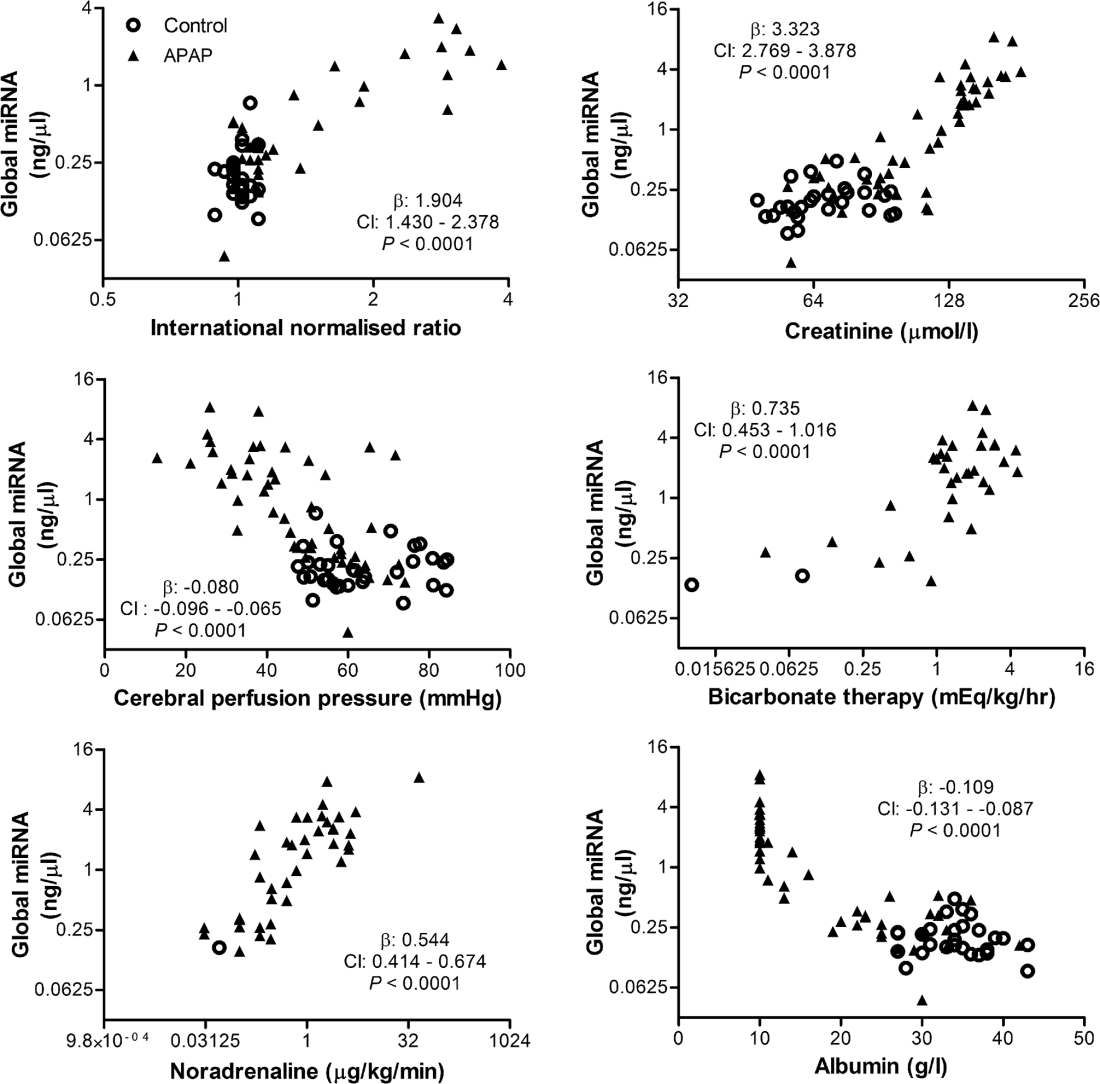
Increasing global miRNA concentrations in plasma associate with parameters of clinical ALF progression. The global miRNA concentrations were compared to six time-matched parameters representing: liver injury; kidney injury; brain injury; acid-base imbalance; cardiovascular stability; vascular permeability. β: fixed effects; CI: confidence interval.

Plasma global miRNA levels (all RNA strands between 10 and 40 nucleotides in length), measured by electropherogram, increased with disease progression in APAP-treated animals, beginning 16h after the onset of APAP dosing and continuing until death (*P* < 0.0001, Fig 1). Global miRNA concentrations were significantly associated with all six markers of disease progression assessed (*P* < 0.0001; Fig 2)

**Fig 3:**
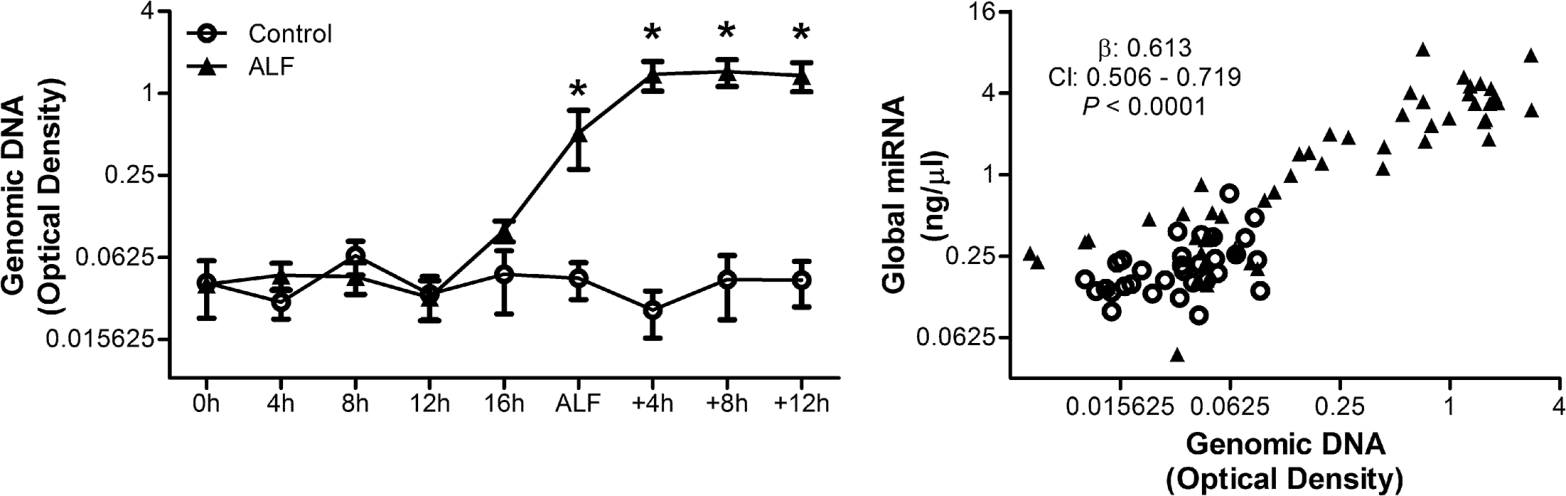
Increasing plasma global miRNA concentrations associate with increasing plasma genomic DNA in APAP-treated animals. Genomic DNA levels were measured in APAP treated pigs (n = 6) and controls (n = 3), and its association with global miRNA concentrations was assessed. Values are means ± SE; * *P* < 0.05 vs. baseline at -20h; β: fixed effects; CI: confidence interval.

To investigate the mechanism of miRNA entering the plasma from tissue, exosomes were isolated from plasma samples and the global miRNA concentration measured. In APAP-treated pigs, as with controls, there was no observable increase in global miRNA concentration in the exosomes (S3 Fig). Whether increasing plasma global miRNA concentrations were a result of tissue damage, was evaluated by measuring plasma levels of an established DAMP, genomic DNA. Plasma genomic DNA levels increased from baseline at ALF (*P* < 0.0001) and continued to rise until death and this increase in genomic DNA was associated with the increase in global miRNA (*P* < 0.0001, Fig 3).

### Tissue-specific miRNA species show the timeline of organ injury in plasma samples

**Fig 4:**
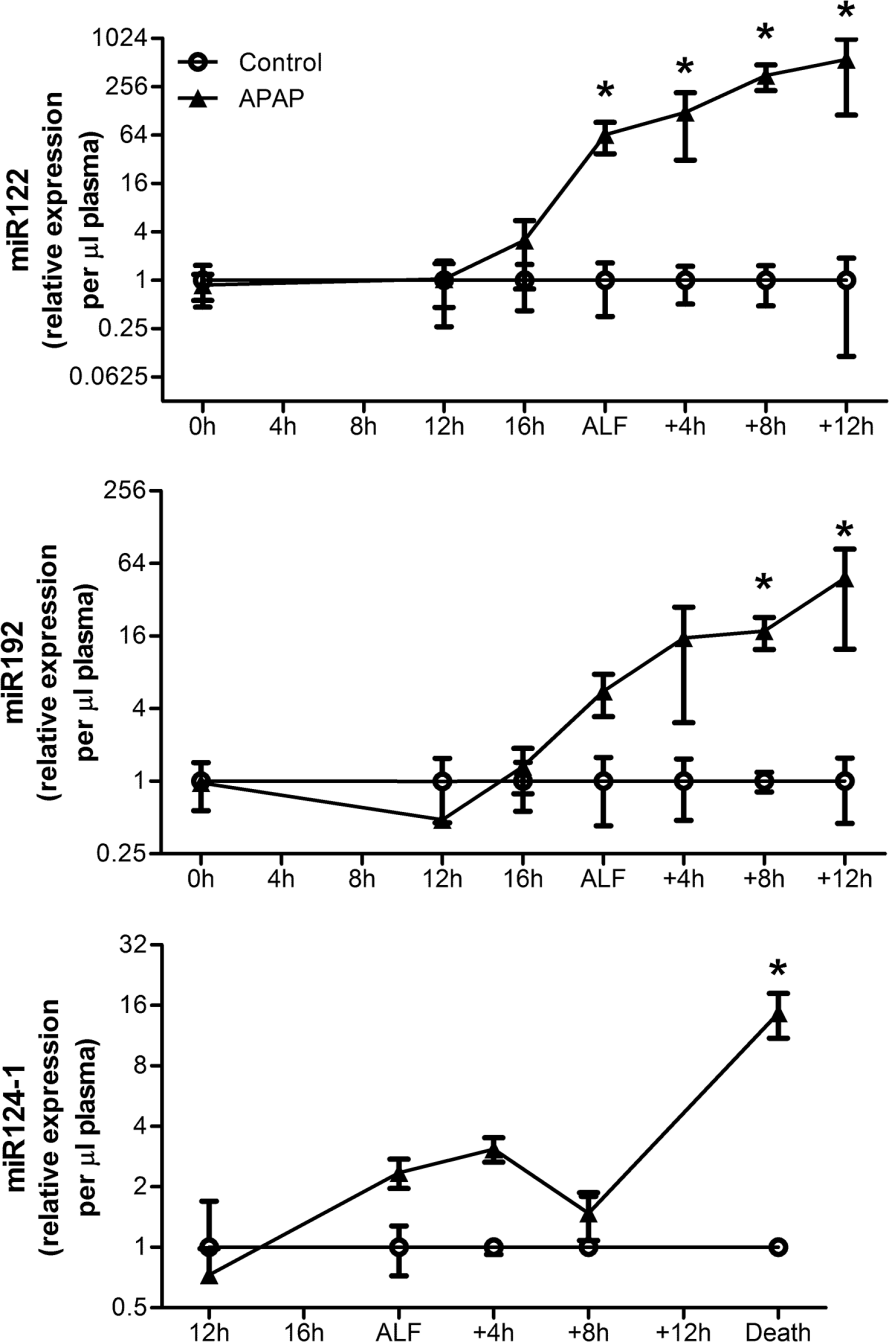
Plasma levels of miR122, miR192 and miR124-1 increase in a time dependent manner in APAP-treated animals. Values are means ± SE of relative expression (2^-ΔΔCt^) for APAP-treated animals (n = 6) to both the endogenous control (miR26a) and the time-matched controls (n = 3); * *P* < 0.05 vs. baseline at -20h.

Plasma levels of miR122, miR192 and miR124-1 were measured by qRT-PCR to assess their contribution to the rise in plasma global miRNA. A time course of increases in these miRNAs was observed in APAP-treated animals (Fig 4). Plasma miR122 levels began to increase prior to ALF and were elevated from the point of ALF to death (*P* < 0.0001), whilst plasma miR192 levels began to increase from the point of ALF and were elevated from 8h after ALF until death (*P* < 0.0001), and plasma miR124-1 levels were only elevated pre-terminally (*P* < 0.0001).

**Fig 5:**
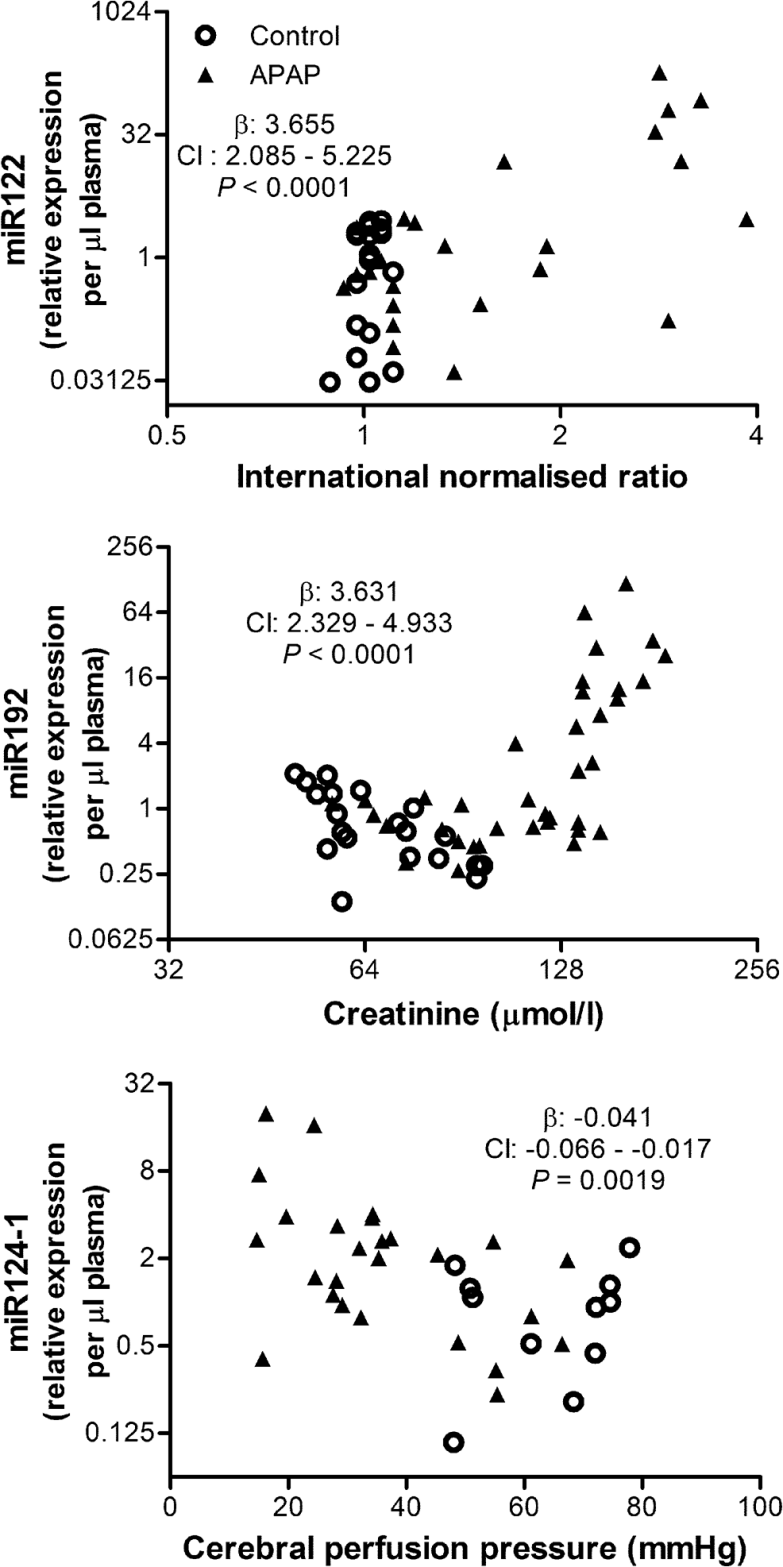
The increase in plasma levels of miR122, miR192 and miR124-1 associate with evidence of liver, kidney and brain injury respectively. Plasma miR122, miR192, miR124-1 and their respective associations with INR, creatinine and CPP. β: fixed effects; CI: confidence interval.

As the time course was suggestive that these miRNA originated from different tissues, their associations with markers of injury of the major organs affected in ALF (liver, kidney and brain) were investigated (Fig 5). Plasma miR122 levels were associated with INR, a marker of liver injury in this model, prior to and including the point of ALF (*P* < 0.0001). The increase in plasma miR192 was associated with increasing creatinine, a marker of kidney injury (*P* < 0.0001). Interestingly, the onset of continuous renal replacement therapy at the point of ALF, mitigated the rise in creatinine, but did not affect the rise in plasma miR192. This was supported by the analysis of the dialysate for miRNA, which revealed that all specific miRNA species tested were in the undetectable range of C_t_s (range: 36.4–39.2 raw C_t_) in the dialysate samples. The terminal rise in miR124-1 was associated with falling CPP, a marker of brain injury in this model (*P* = 0.0019).

### Tissue-specific miRNA species remain unaltered by APAP-induced ALF in tissue samples

**Fig 6:**
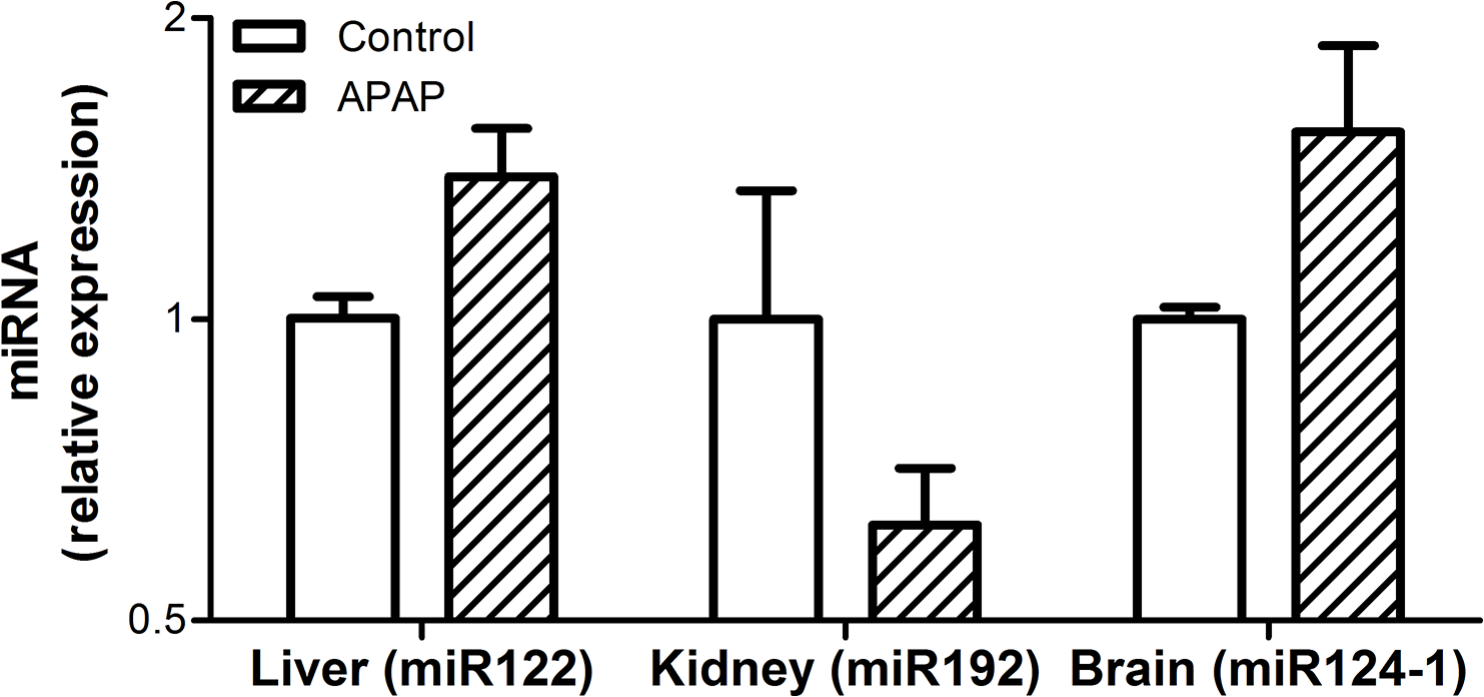
Levels of miR122, miR192 and miR124-1 in their respective tissues. Values are means ± SE of relative expression (2^-ΔΔCt^) for APAP-treated animals to both the endogenous control (miR26a) and the time-matched controls; * *P* < 0.05 vs. baseline at -20h.

The tissue-specific miRNAs, miR122, miR192 and miR124-1, were highly expressed in their respective tissues (range: 15.7–28.4 raw C_t_ and -1.3–1.5 ΔC_t_). Levels of miR122, miR192 and miR124-1 in liver, kidney and brain tissue respectively were not significantly altered by ALF when compared to controls at the end of the study (Fig 6).

## Discussion

This study shows that plasma global miRNA levels increase following APAP administration in pigs and are associated with markers of ALF progression. An increase in plasma global miRNA levels has also been noted (Supplementary information [12]) in a mouse model of APAP-induced ALI. The global miRNA levels lack specificity and may rise in other conditions where there is massive cell injury. In this study, the magnitude of the increase in global miRNA levels probably allowed earlier detection of the ALF associated increase compared to increases of a smaller magnitude in the individual tissue-specific miRNAs. The markers of ALF available in the pig model of ALF used in this study show a clear progression through ALI to ALF, multi-organ failure and death (S2 Fig). Due to the limitations of this model some of the markers are surrogates for those more commonly used. In addition INR was only measured up to the point of ALF as treatment with fresh frozen plasma at ALF would have complicated its interpretation as a marker of liver function. In spite of these limitations, the consistent close association of global miRNA levels to these markers (Fig 2) highlights the ability of global miRNA levels to give an overall indication of ALF progression.

For miRNA to be stable in blood they must either be within an exosome or protein-bound, otherwise they are rapidly degraded [16]. In this study it was observed that isolated exosomes contained only a trace amount of miRNA (S3 Fig), suggesting that the increases in plasma miRNA observed were as a result of increases in protein-bound miRNA. This observation is consistent with a previous study where miR122 was found to be predominantly elevated in the protein-rich fraction in ALF and isolated exosomes during chronic liver injury in mice [34]. In addition analysis of the dialysate revealed that the specific miRNA species were in the undetectable range suggesting that the miRNA must be bound to a sufficiently large protein to prevent passage through the filter membrane. This observation has also been found in another study which investigated plasma and dialysate levels of miR21 and miR210 with different haemodialysis filters [35]. The primary candidate protein would be Ago2 as it is 96kDa, naturally associated with miRNA intracellularly and extracellular levels of Ago2/miRNA complexes have been shown to increase following cell death [17]. Furthermore the release of miRNA into the circulation in the present study was associated with rising levels of plasma genomic DNA (Fig 3). Genomic DNA is a DAMP, which has been shown to rise in humans and rodents following DNA fragmentation caused by APAP metabolite-induced cellular damage [36,37]. Therefore the results of this study suggest that the increase in plasma miRNA occurred due to passive release of protein-bound miRNA in response to cellular damage and injury.

The change in the global miRNA levels highlights the requirement for a robust endogenous control, which does not alter with progression of ALF, for quantification of any organ-specific miRNA. Whilst miR103 was found to be the best endogenous control from six potential genes tested for plasma in a rat model of APAP-induced ALF [38], we are not aware of any published validated endogenous controls for pigs or humans in ALF. MiR26a has previously been identified for use as an endogenous control both in porcine tissues [39] and in human plasma from Hepatitis B virus-infected patients [40]. Here we have validated miR26a for use in porcine plasma and tissue samples, and showed that it is a robust endogenous control for use in ALF samples (Table 1).

The earliest and most marked elevation amongst the specific miRNA species evaluated in this study was seen with miR122 (Fig 4). MiR122 has been described as a liver-specific miRNA, which makes up 70% of the miRNA in hepatocytes [24] and has been shown to be liver-specific in the pig [41]. Circulating miR122 levels increase across a wide spectrum of liver diseases and have been shown to correlate with the severity of liver injury [42,43]. In addition, studies have shown a close association between increasing miR122 and ALT in APAP-induced ALF in mice [12,34]. Since ALT is not a sensitive marker of liver injury in the pig [44] and does not increase with progression of ALF in this model or in other models using pigs [27,45], the close association between increasing miR122 and INR (Fig 5) in this study support the hypothesis that plasma miR122 in porcine ALF originates from the liver. This study provides the first evidence that miR122 is a useful marker of liver injury in porcine liver models.

The second specific miRNA species to increase following ALF was miR192 (Fig 4), which is considered kidney-specific [21] and is highly expressed in the porcine kidney [46]. However it is also expressed in other tissues such as the liver and previous studies have concluded that this was the source of increased plasma miR192 levels in APAP-induced ALF in humans and mice [12,18]. In this study it is clear that plasma miR192 rises more slowly than miR122 and associates with the rise in creatinine (Fig 5). Interestingly creatinine levels pass into the Risk category in the RIFLE criteria and Stage 1 in the AKIN criteria (≥ 1.5x baseline) [47,48] at the point of ALF when miR192 plasma levels begin to rise and reach the Injury category and Stage 2 (≥ 2x baseline) at ALF + 8h (S2 Fig) when the elevation in plasma miR192 becomes significant. The differences in the timeline and the fold-change between miR192 and miR122, the close association with clinically significant changes in creatinine and the high expression of miR192 in the kidneys support the notion that the kidney represents the primary candidate for release of miR192 following the acute kidney injury that occurs during ALF.

The last specific miRNA species to increase was miR124-1 (Fig 4), which has been shown to be enriched in the brain [23,24] and enriched in neurons in pigs [49], and released into the plasma following brain injury in humans and rats [25,26]. In the present model, there is a gradual fall in CPP due to rising ICP following ALF with a rapid increase in ICP pre-terminally. It has been shown in pigs that a CPP of less than 30mmHg corresponds with the brain ischemia threshold (lactate: pyruvate ratio > 30) [50]. Plasma levels of miR124-1 associate with falling CPP (Fig 5) and are significantly elevated when the CPP falls below 30mmHg (S2 Fig), suggesting that the release of miR124-1 into the plasma is likely to be due to relative ischemia in this study. The high levels of miR124-1 found in the brain tissue, support the notion that it originates in the brain. This is the first report of elevated circulating miR124-1 levels as a potential marker of brain injury in ALF.

In this study it was only possible to take biopsies of all three tissues at the end of the study. Having observed a large passive release of each tissue-specific miRNA into the plasma prior to death (Fig 4), a corresponding fall in tissue-specific miRNA expression within in each tissue was expected. However, although there was a trend towards changes in tissue expression, no significant change in tissue expression was observed (Fig 6). The lack of significance may in part be due to the high expression of the tissue-specific miRNA within their respective tissues, when compared to plasma levels, resulting in small fold changes, the small sample size used in this study and the region specific tissue injury caused by APAP, particularly in the liver and kidney. The trend towards an increase in tissue expression of miR122 and miR124-1 within the liver and brain respectively suggests that changes in miRNA expression within the tissues occurred in addition to the passive release evident in the plasma. Therefore miRNAs may play an active role in the response to APAP-induced injury at the tissue level. This is supported by a recent study showing that increased miR122 in hepatocytes resulted in down-regulation of target genes, which impair liver regeneration, and was associated with an increased chance of survival in ALF patients [20]. In light of this, targeted investigation of changes in miRNA expression in each tissue during the evolution of its injury is warranted to gain insights into their mechanisms of action and whether they may represent therapeutic targets.

Since there is no physical way of identifying the tissue of origin of a specific miRNA species, studies that investigate time courses and associations between markers, such as this one, can give a strong indication of their likely origin. In addition, identifying a time course of tissue injury during ALF progression facilitates further tissue-specific mechanistic studies and could, in future, enable the identification of therapeutic windows and targeted interventions. Since miRNAs do not represent current treatment targets, unlike more commonly used clinical markers, and appear to be more closely linked to tissue injury rather than function, they may facilitate assessments of interventions that target these common clinical markers. So, whilst this study cannot provide causal relationships, it does elucidate the potential utility of miRNA when investigating the multi-organ injury occurring during ALF.

In summary, we have shown for the first time that an increase in plasma global miRNA levels may be detected prior to increases in individual specific miRNA species and that this increase occurs in association with evidence of cellular damage, and clinical and analytical parameters of ALF progression. Furthermore by quantifying individual tissue-specific miRNA species, we were able to visualise the timeline of organ injury in this model, starting with the liver at the point of ALF, followed by the kidney and finally the brain pre-terminally. It is hoped that this study will form a foundation from which to further investigate the role of miRNA in tissue injury in ALI and ALF.

## Supporting Information

S1 Fig Assessment of the potential endogenous controls. (PDF)

S2 Fig The six markers of ALF progression from the porcine model. (PDF)

S3 Fig Mechanism of release of global miRNA into the circulation. (PDF)

S1 Table Details of miRNA sequences and assays. (PDF)
