# Supplementary material for: Circulating microRNAs Reveal Time Course of Organ Injury in a Porcine Model of Acetaminophen-Induced Acute Liver Failure
Source: PLoS One. 2015 May 27;10(5):e0128076. doi: 10.1371/journal.pone.0128076 (PMC4446266; doi:10.1371/journal.pone.0128076)
Supplement: S1 Fig — (PDF) [file pone.0128076.s001.pdf]

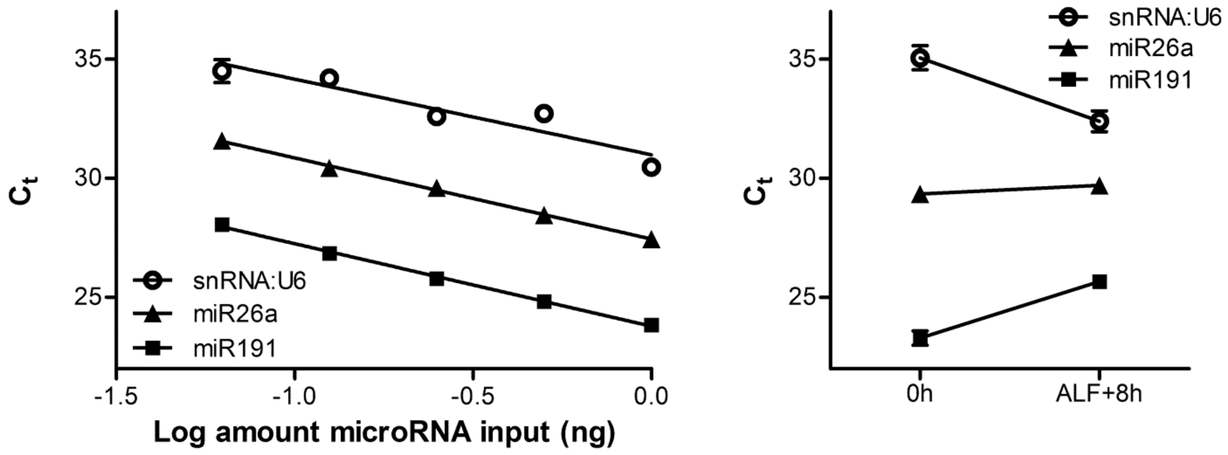

S1 Fig. Assessment of the potential endogenous controls. The potential endogenous controls, snRNA:U6, miR26a and miR191, were assessed for amplification efficiency across a range of input miRNA amounts (0.0625 – 1ng). In addition their stability with progression of ALF was assessed using APAP-treated animals (n=3), the comparison of baseline samples at 0h with the diseased samples at ALF + 8h is shown. Values are means ± SE; Ct, threshold cycle.
