# Supplementary material for: Circulating microRNAs Reveal Time Course of Organ Injury in a Porcine Model of Acetaminophen-Induced Acute Liver Failure
Source: PLoS One. 2015 May 27;10(5):e0128076. doi: 10.1371/journal.pone.0128076 (PMC4446266; doi:10.1371/journal.pone.0128076)
Supplement: S2 Fig — (PDF) [file pone.0128076.s002.pdf]

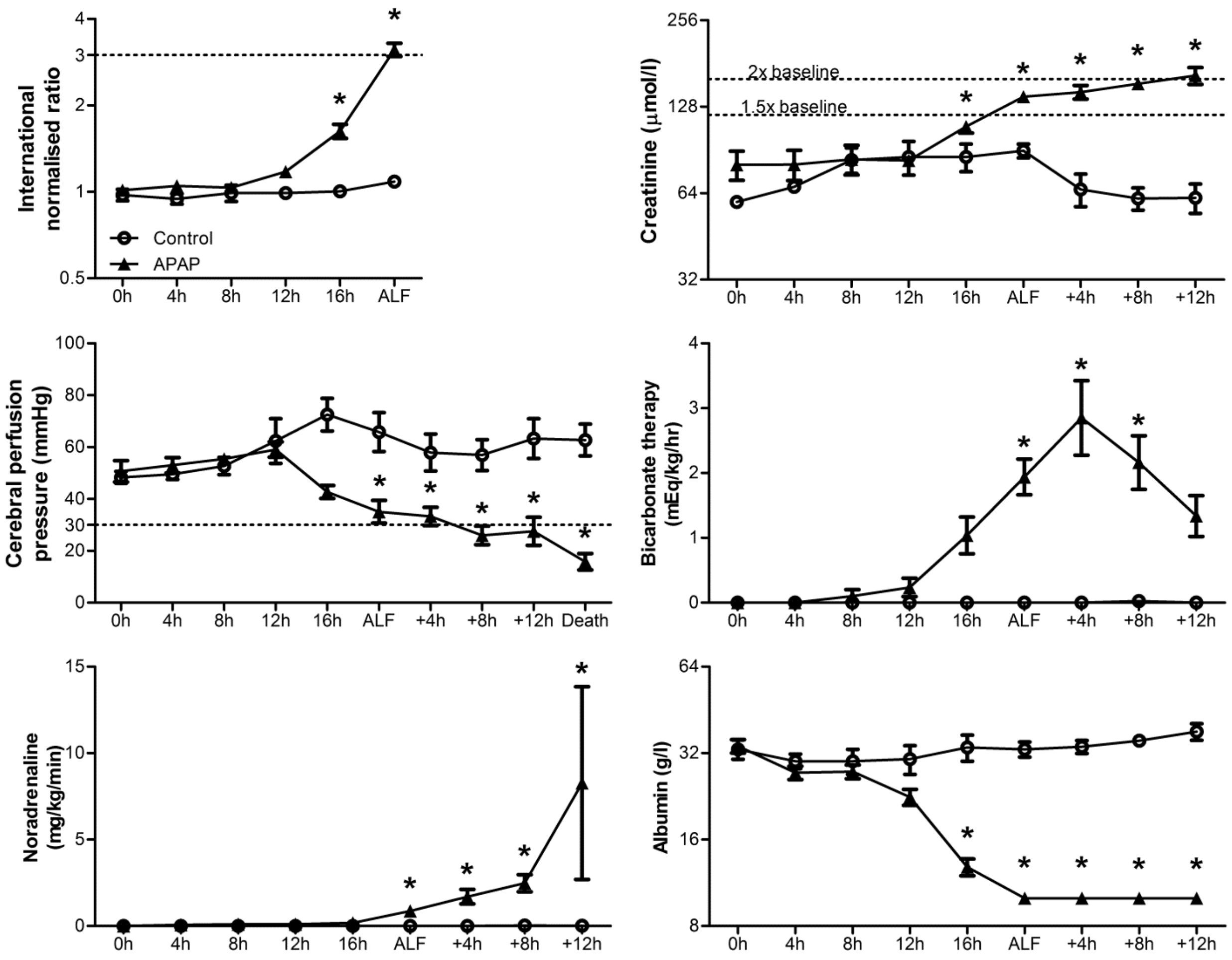

S2 Fig. The six markers of ALF progression from the porcine model. International normalised ratio, creatinine, cerebral perfusion pressure, bicarbonate therapy, noradrenaline therapy and albumin representing liver injury, kidney injury, brain injury acid-base imbalance, cardiovascular stability and vascular permeability respectively, reproduced in graphical form from Lee *et al* (2013). Values are means ± SE; * $P < 0.05$ vs. control animals.
