# Supplementary material for: Circulating microRNAs Reveal Time Course of Organ Injury in a Porcine Model of Acetaminophen-Induced Acute Liver Failure
Source: PLoS One. 2015 May 27;10(5):e0128076. doi: 10.1371/journal.pone.0128076 (PMC4446266; doi:10.1371/journal.pone.0128076)
Supplement: S3 Fig — (PDF) [file pone.0128076.s003.pdf]

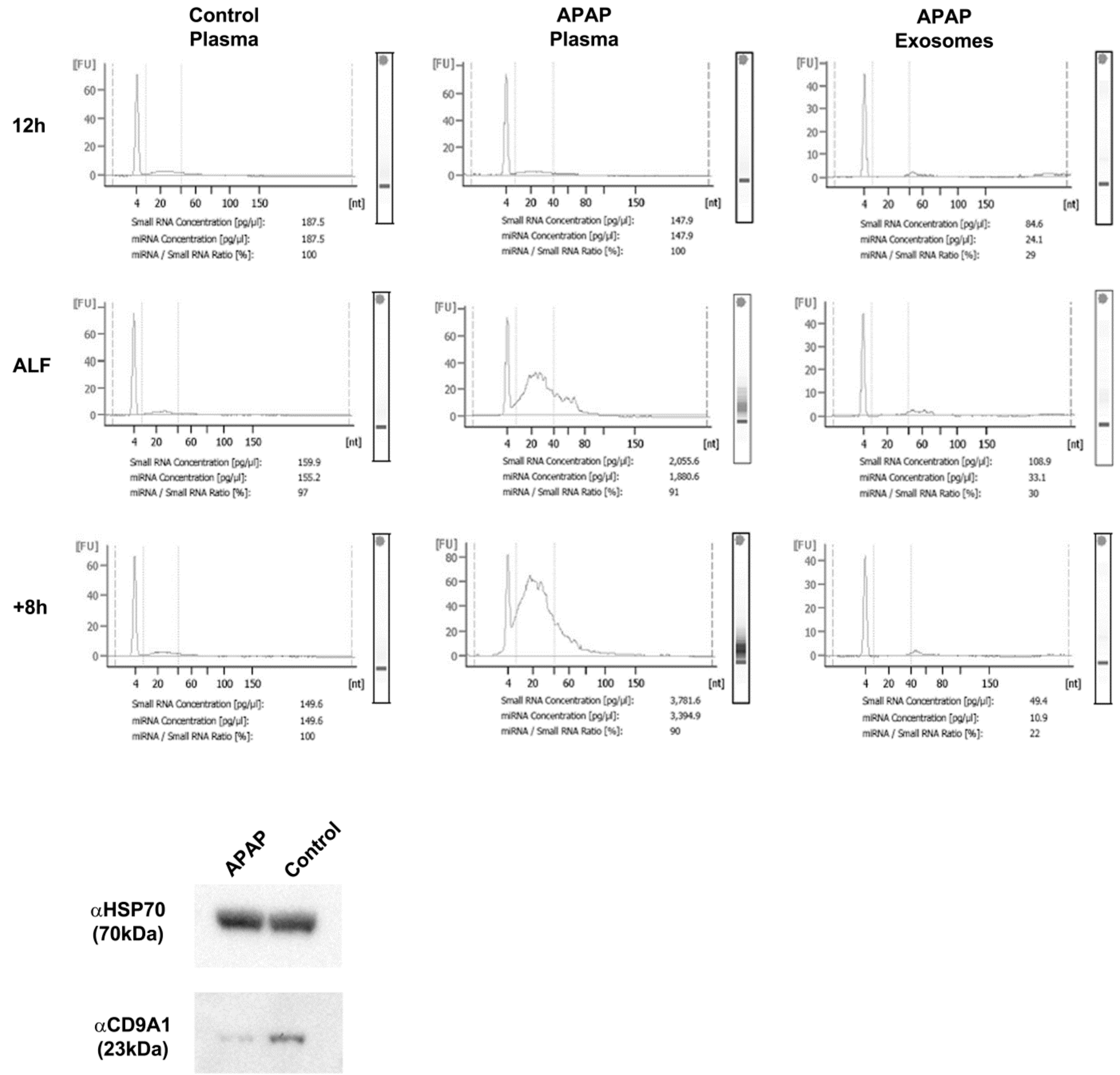

S3 Fig. Mechanism of release of global miRNA into the circulation. Circulating exosomes were isolated from plasma samples and the fraction was checked for the presence of exosome "marker" proteins. Electropherogram traces for whole plasma from one representative control animal and one representative APAP animal, and for isolated exosomes from the same APAP animal. Western blot showing the presence of exosome "marker" proteins (αHsp70 and αCD9A1) following exosome isolation, two representative samples.
