# Supplementary material for: Circulating microRNAs Reveal Time Course of Organ Injury in a Porcine Model of Acetaminophen-Induced Acute Liver Failure
Source: PLoS One. 2015 May 27;10(5):e0128076. doi: 10.1371/journal.pone.0128076 (PMC4446266; doi:10.1371/journal.pone.0128076)
Supplement: S1 Table — (PDF) [file pone.0128076.s004.pdf]

S1 Table. Details of miRNA sequences and assays. Names, RNA sequences, accession numbers and TaqMan MicroRNA Assay numbers (Applied Biosystems, Foster City, CA) for all the small RNA and miRNA used.

Gene	Sequence	Accession Number	Assay number (if inventoried)
**snRNA:U6**	GTGCTCGCTTCGGCAGCACATA	EU520423 & X59362*	Customised
**miR26a**	UUCAAGUAAUCCAGGAUAGGCU	MI0002429	000405
**miR191**	CAACGGAAUCCCAAAAGCAGCUG	MI0013095	002299
**miR122**	UGGAGUGUGACAAUGGUGUUUGU	MI0002413	000445
**miR192**	CUGACCUAUGAAUUGACAGCC	MI0013127	000491
**miR124-1**	UAAGGCACGCGGUGAAUGCCA	MI0010680	000239

* Porcine snRNA:U6 is not fully sequenced, so by aligning the partial porcine sequence available with the human snRNA:U6 sequence (BLAST, NCBI, Bethesda, MD), a 22 nucleotide sequence was identified from which a customised assay was made (Applied Biosystems).
